# Bilateral Tibial Tubercle Avulsion Fractures With an Associated Patellar Tendon Avulsion in an Adolescent Patient

**DOI:** 10.5435/JAAOSGlobal-D-22-00105

**Published:** 2023-09-15

**Authors:** James H. Dove, Giancarlo Medina Perez, Alexandre Boulos, Craig P. Eberson

**Affiliations:** From the Department of Orthopedic Surgery, Warren Alpert Medical School of Brown University (Dr. Dove, Dr. Boulos, and Dr. Eberson), and the Warren Alpert Medical School of Brown University, Providence, RI (Medina Perez).

## Abstract

Tibial tubercle fractures in pediatric patients are increasing in frequency as more children participate in sports. These injuries are often seen in boys engaging in jumping activities before closure of their proximal tibial physis. Bilateral tibial tubercle fractures have been reported in the literature, but less frequent are associated patellar tendon ruptures with fracture of the tubercle. In this case report, we present an 11-year-old girl who sustained bilateral tibial tubercle fractures, including an associated patellar tendon rupture from the tubercle on the right lower extremity. We describe our technique for the management of both injuries, which included a primary patellar tendon repair for the right leg and Kirschner wire fixation of the displaced tubercle for the left leg. The patient ultimately had a successful outcome at the final follow-up with healed fractures and full range of motion of both knees. In this case report, we also present similar cases from the literature and the differing treatment strategies.

Historically, tibial tubercle avulsion fractures have been deemed uncommon injuries in adolescents comprising between 0.4% and 2.7% of all epiphyseal injuries and less than 1% of all physeal injuries.^1,2^ These injuries are more commonly seen in boys from 12 to 16 years after jumping activities and occur because of the pattern of closure in the proximal tibial physis, which is distally in the posteromedial to anterolateral direction, making the tubercle susceptible to avulsion injury.^2-4^ Bilateral tibial tubercle avulsion fractures are extremely rare with only 25 reported cases in the literature.^5-7^ In addition, tibial tubercle avulsion fractures with concomitant patellar tendon rupture from the tibial tubercle have only a few case reports involving children.^8,9,10,11^

In this case report, we present a unique case involving an 11-year-old girl who sustained bilateral tibial tubercle avulsion fractures with an associated patellar tendon rupture from the tubercle on the right lower extremity. She underwent patellar tendon repair on the right and fixation of the tibial tubercle avulsion fracture on the left. The patient and her parents were informed that data concerning the case would be submitted for publication, and they provided consent.

## Case Report

**Figure 1:**
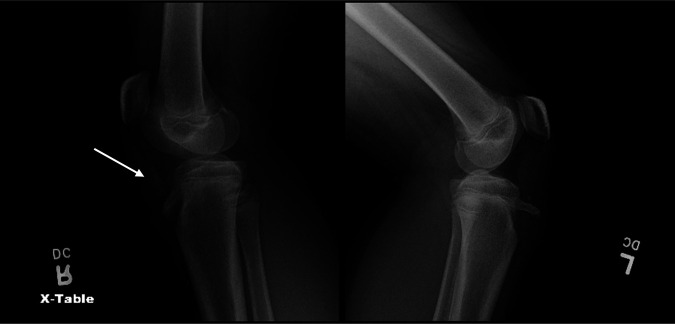
Radiographs of the right and left knees that reveal bilateral tibial tubercle fractures. The right knee reveals an ossific fragment proximal to the tibial tubercle (arrow).

**Figure 2:**
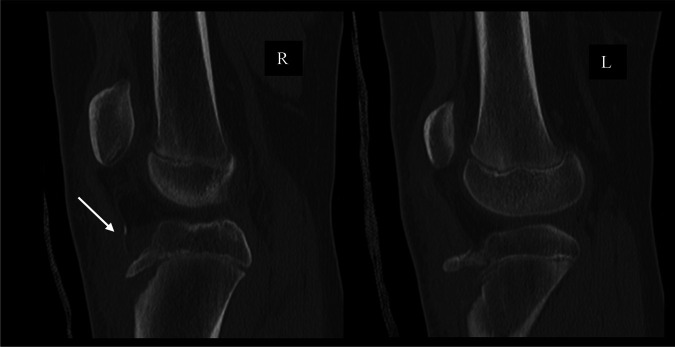
CT scan of bilateral knees did not reveal any intra-articular involvement of the injury. The tibial tubercle sleeve is better appreciated on this sagittal cut of the right knee (arrow).

The patient initially presented to the emergency department after a gymnastics injury. She recalled attempting a front flip off a trampoline but landed awkwardly and had immediate pain to both knees. Radiographs obtained in the emergency department revealed bilateral displaced tibial tubercle fractures, as shown in Figure 1. Closer inspection of the radiograph of the right knee revealed an ossific fragment or sleeve fracture of the tibial tubercle. On her physical examination, the patient had tenderness and swelling present about both knees. She was unable to do a straight leg raise for either leg. A CT scan was then performed on both knees, as shown in Figure 2. The patient was later admitted and closely monitored overnight with plans for surgery the next day.

In the operating room, the patient was placed supine on a radiolucent table with high bilateral thigh tourniquets. After induction of general anesthesia, prophylactic antibiotics were given and the patient was prepped and draped in the usual sterile fashion.

### Right Knee

A midline incision was made over the anterior knee from the distal pole of the patella to approximately 3 cm below the tibial tubercle. Dissection was done through the subcutaneous tissue until fracture and hematoma were identified. At the fracture site, the patellar tendon avulsion from the tubercle was identified with a small cartilaginous rim. There was substantial plastic deformation of the tubercle fragment. The tubercle fragment was reduced into its bed with no intervening soft tissue; however, fluoroscopy showed persistent deformity. The decision was made to perform the patellar tendon repair directly to the tibia without fixation of the tubercle fracture to avoid further risking growth disturbance from the tubercle fragment.

A double-row technique was used for the repair of the patellar tendon avulsion. One #2 FiberWire was sewn in a Krackow fashion up and down the central third of the tendon. A second #2 FiberWire was sewn in a Krackow fashion up and down the lateral and medial edges of the tendon. Two tibial transosseous tunnels were made. The suture from the central third of the tendon was placed and tied through the proximal tunnel. The suture from the medial and lateral edges were placed and tied through the distal tunnel. The repair helped to further reduce the displaced tubercle fragment. After the repair, the knee was bent 90° without gapping of the repair. The peritenon was repaired over the tendon, and a layered closure was performed. The tourniquet was deflated, and attention was turned to the left knee.

### Left Knee

The same opening approach was used on the left knee. At the fracture site, the patellar tendon was found to be attached to the tubercle fragment and the tubercle had a partial-thickness (greenstick)-type fracture. A short flap of the tendon remained attached distally, contiguous to the periosteum of the tibia.

**Figure 3:**
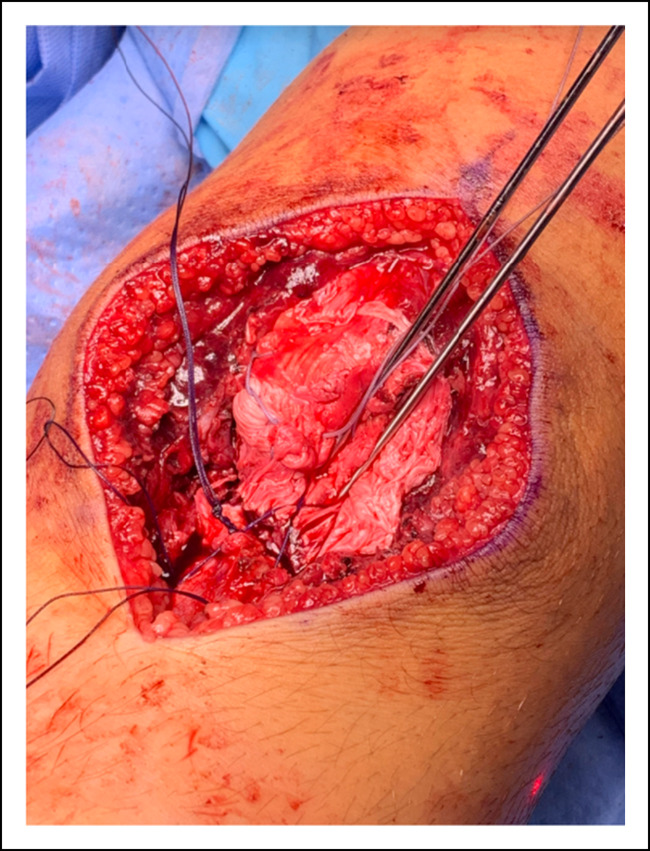
Image showing two Kirschner wires holding the displaced tubercle in position, and reduction was secured with a tension band technique using #1 Vicryl suture through a transosseous tunnel.

Similar to the right knee, the distal tibial tubercle fragment was anteriorly displaced and flipped upwardly. Given that the fracture was anchored firmly at its proximal end, the tubercle fragment was reduced into its bed successfully with posteriorly directed pressure. Rather than using screw fixation, a tension band technique was chosen to minimize transphyseal fixation. Two oblique Kirschner wires were driven to the back cortex of the tibia, but not through it. A #1 Vicryl suture was then placed in a figure-of-8 fashion through the drill hole and tied tightly securing the reduction as shown in Figure 3. Pins were then cut, bent, and impacted against the anterior tibia distal to the joint. The pins were well-covered by the patellar tendon. Dissolvable suture was chosen so resorption would occur after healing and not represent a permanent tether anterior to the tubercle physis. A #2 FiberWire was sewn in a Krackow fashion up and down the central third of the patellar tendon for reinforcement and tied through a tibial transosseous tunnel. This helped to further stabilize the tubercle fragment.

**Figure 4:**
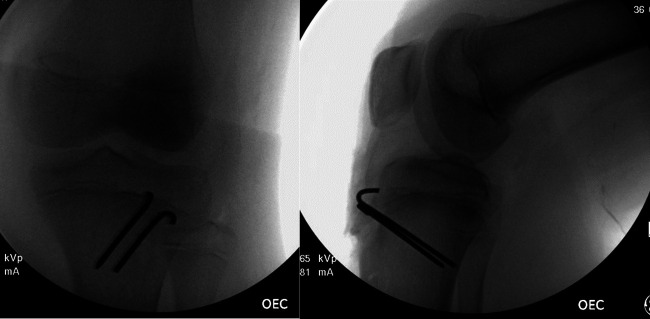
Intraoperative films of left knee demonstrating stability of tibial tubercle fracture with flexion of the knee following fixation with Kirschner wires.

Once again, at the end of the repair, the knee was flexed 90° with stability of the fracture fragment and patellar tendon as seen in Figure 4. The surgical wound was then closed in the same approach as the right knee. After the procedures, both legs were placed in long leg casts in extension, which were bivalved to allow for swelling.

## Discussion

Bilateral tibial tubercle fractures in adolescents are rare injuries with only approximately 25 case reports in the literature.^5-7^ Even rarer is the instance of an associated patellar tendon rupture from the displaced tibial tubercle. Mosier and Stanitski^10^ found that extensor mechanism disruption in tibial tubercle fractures is associated with comminuted tubercle fragments, classified as Ogden type B injury. Based on the Ogden classification and subsequent modification by Ryu et al, our patient presented with a combination of a type IIB and type V injury on the right knee and a type IIB injury on the left knee.^12,13^

Given the displaced nature of the fracture, surgical treatment was indicated to restore the integrity of the extensor mechanism. Owing to the pull of the patellar tendon, displaced fractures treated nonsurgically are at high risk of persistent fracture displacement and poor fracture healing.^4^ Most commonly, internal fixation is achieved with cancellous, partially threaded screws in skeletally mature patients and smooth Kirschner wires are used for patients >3 years from skeletal maturity.^2,3^ In the case of the patient presented, a patellar tendon repair was performed on the right knee with no screw or wire to prevent additional risk of growth disturbance from the tubercle fragment. The correct anatomic relationship between the patellar tendon and the tibia was established with direct repair of the tendon to the tibia. The left knee tibial tubercle fragment was reduced using Kirschner wires, and a tension band technique was used with absorbable suture. This approach was selected based on her young age to minimize the size of the implants crossing the physis.

Displaced tibial tubercle fractures with associated patellar tendon avulsions are rare in adolescents with no definitive surgical technique. In a 13-year-old adolescent girl with this presentation, Behery et al^9^ described their repair which involved reattachment of the tendon through a tibial transosseous tunnel. The repair was supplemented with FiberWire that passed through a patellar tunnel into a more distal tibial tunnel,^9^ similar to techniques used in adults. In the case of our patient, two tibial tunnels were used, one more proximal than the other. By tying suture from the central third of the tendon through the proximal tunnel and suture from the medial and lateral edges through the distal tunnel, the patellar tendon was well-approximated, and the repair was robust enough that the decision was made not to add a patellar tunnel for reinforcement.

When comparing patients with bilateral versus unilateral tibial tubercle avulsion fractures, Fernandez et al and Kushare et al^14^ found no notable differences in functional outcomes between the two patient groups.^5,14^ However, Kushare et al^14^ found a higher complication rate (63% versus 21%) in the bilateral tibial fracture group compared with the unilateral tibial fracture group. The most common complication in the bilateral group was implant removal (4/7) and wound dehiscence (2/7).^14^

**Figure 5:**
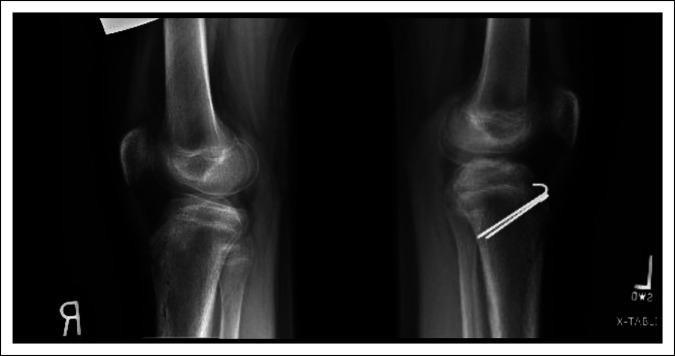
Radiograph of the right knee reveals healing of the tibial tubercle fracture and that of the left knee reveals appropriate positioning of Kirschner wires with healing of the tibial tubercle fracture. Proximal and distal tibial tunnels can be appreciated in the right knee.

Most commonly, in unilateral tibial tubercle fractures, a patient's leg is immobilized in a long leg cast for 4 weeks with partial weight-bearing, followed by physical therapy and a hinged knee brace.^2^ For the patient presented in this case, radiographs at 6 weeks after surgery revealed appropriate healing in both knees, as shown in Figure 5.

Given the difficulty of partial weight-bearing in bilateral injuries, range of motion was initiated on the left, fractured side at week 4 and weight-bearing initiated in a hinged brace at 6 weeks. The right side with the associated tendon avulsion was progressed more slowly, with motion initiated at 6 weeks and weight-bearing in a brace at 8 weeks. Braces were discontinued at 3 months, with full motion obtained in both knees.
